# BCG overexpressing Ag85B reprograms host immune response through coordinated transcriptional networks

**DOI:** 10.3389/fimmu.2026.1876830

**Published:** 2026-08-12

**Authors:** Vivek Chauhan, Rakesh Kumar, Raja Veerapandian, Enrique I. Ramos, Chinnaswamy Jagannath, Shrikanth S. Gadad, Subramanian Dhandayuthapani

**Affiliations:** 1 Center of Emphasis in Infectious Diseases, Department of Molecular and Translational Medicine, Paul L. Foster School of Medicine, Texas Tech University Health Sciences Center El Paso, El Paso, TX, United States; 2 South Texas Center of Excellence in Cancer Research and Department of Medicine and Oncology, School of Medicine, The University of Texas Rio Grande Valley, Edinburg, TX, United States; 3 Department of Biology, University of Texas El Paso, El Paso, TX, United States; 4 Department of Pathology and Genomic Medicine, Houston Methodist Research Institute & Weill Cornell Medical College, Houston, TX, United States; 5 Center of Emphasis in Cancer, Department of Molecular and Translational Medicine, Paul L. Foster School of Medicine, Texas Tech University Health Sciences Center El Paso, El Paso, TX, United States

**Keywords:** Ag85B, antigen presentation, cytokine signaling, immunometabolism, M. tuberculosis, recombinant BCG, RNA-seq, T-cell differentiation

## Abstract

Antigen 85B (Ag85B), an immunodominant protein secreted by *Mycobacterium tuberculosis* (Mtb), induces robust T-cell-mediated immunity in host cells. Previous studies have demonstrated that recombinant BCG strains overexpressing Ag85B (BCG85B) confer significant protection against tuberculosis in mouse models. This study hypothesized that enhanced protection by recombinant BCG85B results from modulation of host immune gene expression by the Ag85B antigen. To test this, RNA-seq analysis was performed on splenocytes from mice immunized with the wild-type BCG (BCG-WT) and BCG85B strains. Differentially expressed genes (DEGs) in the splenocytes of BCG85B-immunized mice indicated upregulation of immune-related KEGG pathways, including Th1/Th2 and Th17 differentiation, NF-kappa B signaling, and cytokine-cytokine receptor interactions, as well as Gene Ontology (GO) processes such as immune response activation, α-β T cell activation, T-cell production and differentiation, IL-15 mediated signaling, and cytokine-mediated signaling pathways and down regulation of metabolic and digestive processes. Additionally, transcription factor (TF) and protein-protein interaction (PPI) analyses confirmed upregulation of genes involved in T cell receptor signaling and immune activation (*Cd5*, *Zap70*, *Icos*, *Ctla4*, *Gzmb*, *Cd3e*, and *Il7r*) in splenocytes from BCG85B-immunized mice. Collectively, these findings indicate that Ag85B overexpression enhances adaptive immune activation and reprograms cellular metabolic pathways, thereby increasing vaccine efficacy.

## Introduction

Tuberculosis (TB), caused by *Mycobacterium tuberculosis* (Mtb), remains one of the most prevalent infectious diseases worldwide despite extensive research and control efforts. In 2024, the global incidence of TB was estimated at 10.7 million cases, resulting in approximately 1.23 million deaths (1). A substantial proportion of these cases involve drug-resistant TB (DR-TB) and TB with HIV coinfection (HIV-TB), both of which present significant challenges due to high treatment costs and persistently low success rates. The Bacillus Calmette-Guérin (BCG) vaccine is currently the only approved vaccine for TB; however, its protective efficacy diminishes over time and does not prevent adult pulmonary TB (2, 3). Consequently, multiple research groups are pursuing novel vaccine strategies, including subunit vaccines based on Mtb protein antigen, naked DNA vaccines encoding Mtb antigenic genes, killed mycobacteria, live attenuated mycobacteria, and live attenuated mycobacteria or viruses expressing Mtb antigens, and, more recently, mRNA vaccines targeting Mtb antigenic genes (4–8).

One approach to developing alternative or novel TB vaccines is to use Mtb secretory proteins or their corresponding genes to enhance immunogenicity (9, 10). This strategy leverages the immunodominant properties of these proteins, which enable them to elicit robust immune responses in the host. While several secretory proteins such as ESAT-6, CFP-10, Ag85B, MPT64, MPT83, PE, and PPE have been investigated (11), antigen 85B (Ag85B) of Mtb, encoded by the *fbpB* or *Rv1886c* gene, remains the most extensively studied (12–19). Ag85B was initially identified as one of three fibronectin-binding proteins (Fbps), along with Ag85A (FbpA) and Ag85C (FbpC), which together form the Ag85 complex (20, 21). All three proteins are approximately 30 kDa in size and possess mycolyl transferase activity, which contributes to cord-factor formation and cell wall biosynthesis (20). Beyond their fibronectin-binding and enzymatic functions, the Ag85 complex proteins are highly immunogenic and can induce both T-cell and B-cell responses in the host (20, 21). Ag85B is secreted early during infection and is efficiently recognized by both CD4^+^ and CD8^+^ T cells (20, 21), thereby producing Th1-type immune responses characterized by the secretion of IFN-γ and TNF-α, which are essential for host control of intracellular mycobacteria (19, 22).

Numerous studies have evaluated Ag85B as a subunit vaccine candidate against tuberculosis, and most have combined it with other secretory or non-secretory proteins (12–19). Several of these combinations demonstrated strong protective effects in preclinical studies, and vaccine candidates developed by the Andreson group by fusing Ag85B-TB10.4 (hybrid 4, or H4) and Ag85B-ESAT6-Rv2660c (hybrid 56, or H56) with the IC31 adjuvant have progressed to clinical trials (23, 24). In addition to subunit vaccines, the *fbpB* gene encoding Ag85B has been incorporated into DNA vaccines (25) and viral-vectored vaccines (26, 27). Recently, Ag85B has been evaluated as a mRNA vaccine against TB and other mycobacterial infections (28, 29). Furthermore, *fbpB* has been utilized in the development of recombinant BCG vaccines. Horwitz and colleagues first constructed BCG30, which overexpresses Ag85B, and demonstrated significantly enhanced immunity against tuberculosis in an animal model (30). Our group also constructed a similar BCG strain, BCG85B, and demonstrated that the increased protection conferred by BCG overexpressing Ag85B was due to its ability to induce autophagy in antigen-presenting cells (APCs) (31). We also have demonstrated that the protective effects of Ag85B can be enhanced by fusing Ag85B to a TLR-2 activating peptide C5 and overexpressing it in BCG (BGC85C5) (32).

Although Ag85B has been incorporated into various vaccine strategies, the cellular mechanisms by which it modulates host responses remain poorly understood. In particular, the effects of Ag85B on early transcriptional responses, cytokine secretion, antigen presentation pathways, and intracellular signaling remain to be elucidated. Comparative host-cell response profiling, which systematically compares how host cells respond to a pathogen or stimulus, has proven highly informative for understanding host-pathogen interactions (33–35). In particular, transcriptomics by RNA sequencing (RNA-seq) is a powerful high-throughput technology that enables genome-wide, quantitative profiling of the transcriptome, capturing both known and novel RNA species (36, 37). Additionally, by revealing alternative splicing, non-coding RNAs, and differential gene expression, RNA-seq provides a more comprehensive view of transcriptional regulation than older methods (36, 38). Transcriptomic analyses of epithelial or immune cells infected with different bacterial strains, including Mtb, have revealed pathogen-specific immune signatures and pathway-level differences in cytokine induction, antigen presentation, and metabolic remodeling (39–41). Thus, the goal of the study was to apply transcriptomic analysis to elucidate host cellular responses to Ag85B. We used the BCG overexpressing Ag85B (BCG85B) in this study because this strain and its corresponding BCG30 have shown significant protective effects against TB in a mouse infection model (30, 31).

## Results

### BCG85B modulates host gene expression in comparison to BCG-WT

**Figure 1:**
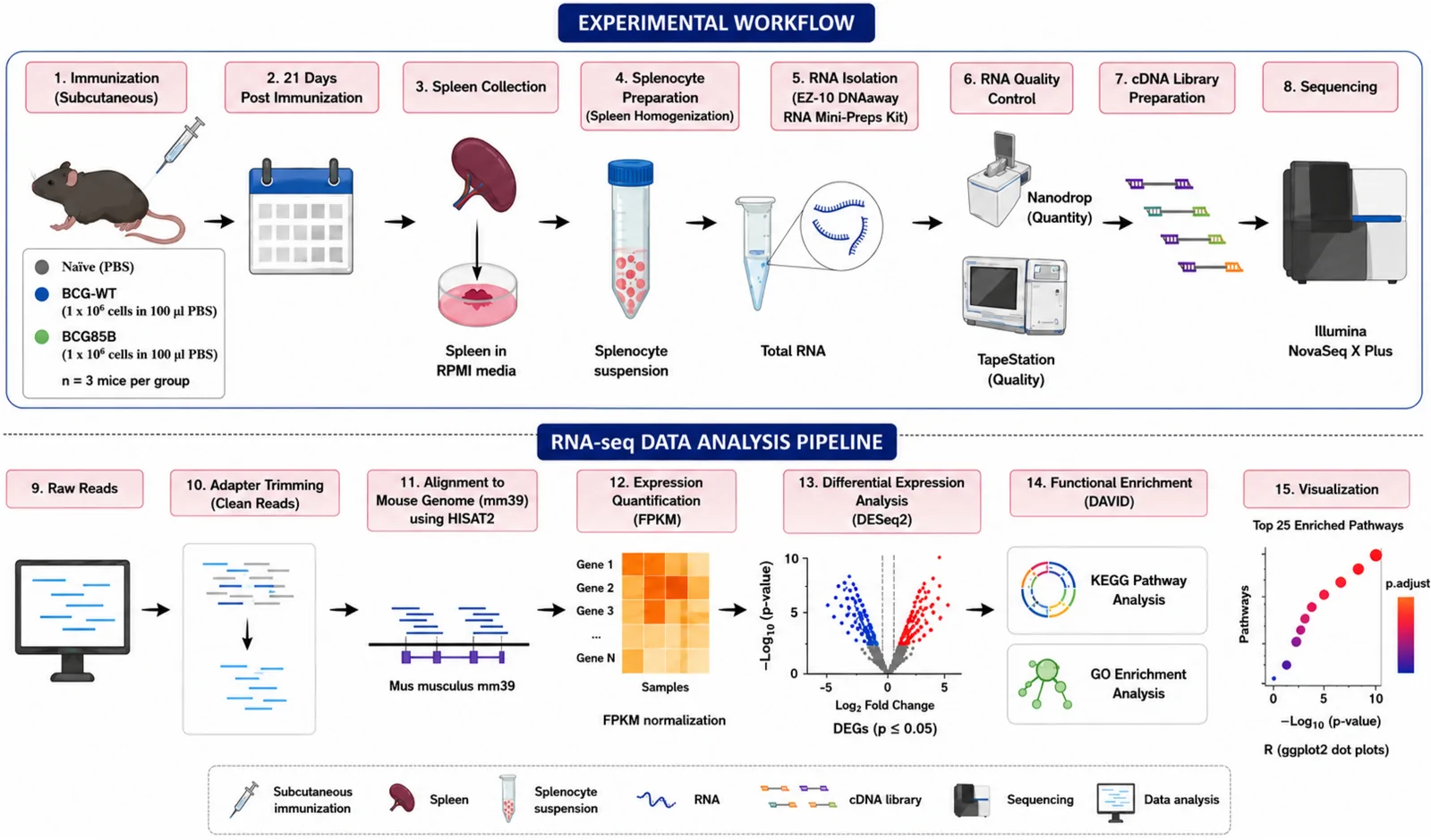
Schematic overview of experimental design and RNA-seq analysis pipeline. C57BL/6J mice were immunized subcutaneously with either BCG-WT or BCG85B (1 × 10^6 CFU in 100 µl PBS), while control (naïve) mice received PBS alone (n = 3 per group). At 21 days post-immunization, mice were euthanized, and spleens were aseptically collected. Spleen tissues were homogenized to obtain splenocyte suspensions, followed by total RNA isolation using the EZ-10 DNAaway RNA Mini-Preps Kit. High-quality RNA samples were used for cDNA library preparation and sequenced on the Illumina NovaSeq X Plus platform. Raw sequencing reads were processed to remove adapter sequences and low-quality bases, yielding clean reads. The filtered reads were aligned to the *Mus musculus* reference genome (mm39) using HISAT2. Gene expression levels were quantified and normalized as fragments per kilobase of transcript per million mapped reads (FPKM). DEGs were identified using the DESeq2 package in R based on statistical significance (p ≤ 0.05). Thereafter, functional enrichment analysis of DEGs was performed using the DAVID bioinformatics platform. Image was prepared using BioRender.

**Figure 2:**
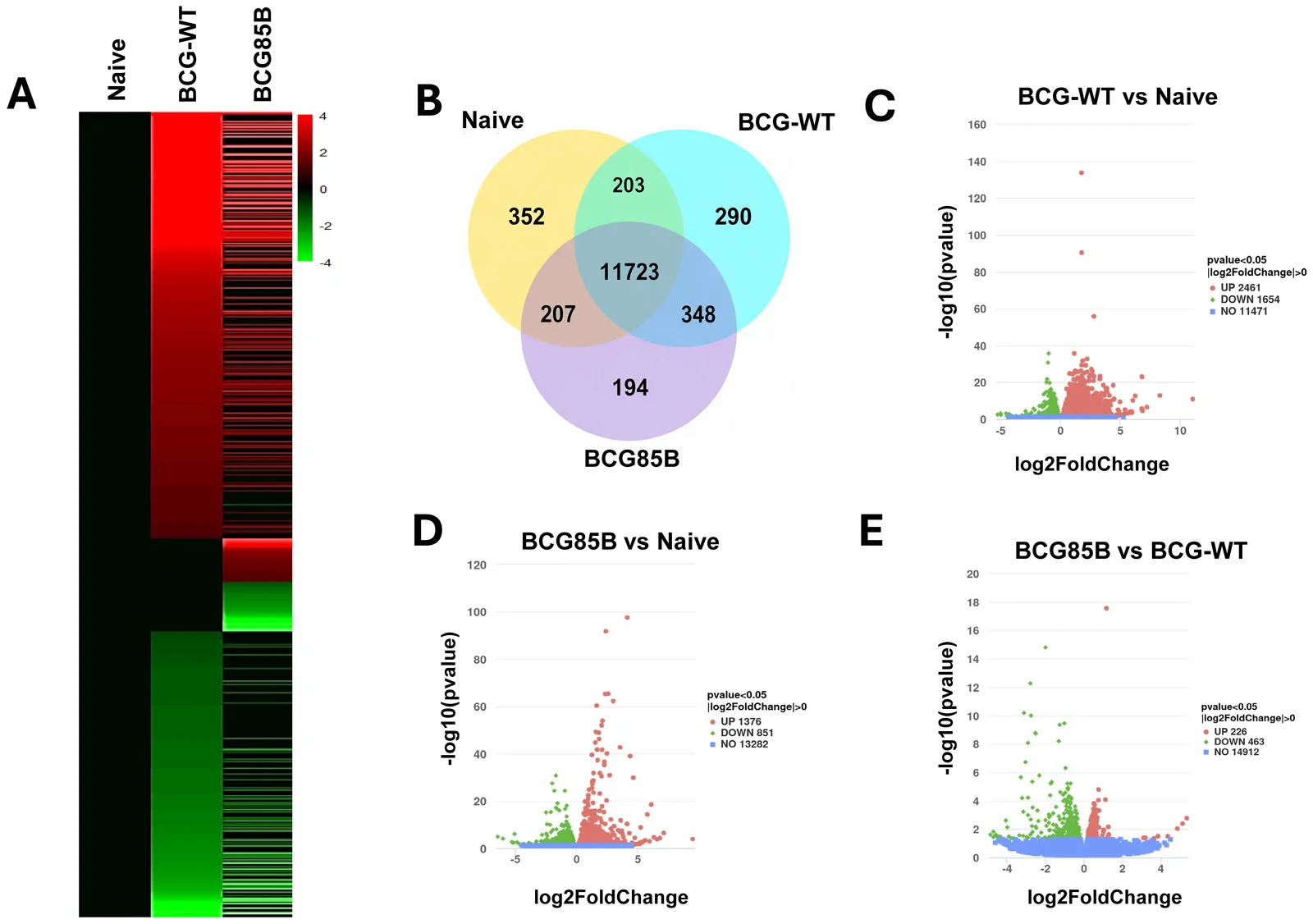
Differential gene expression (DEGs) profile based on RNA-seq analysis of mice splenocytes after infection with BCG-WT and BCG85B. **(A)** Heatmap illustrating the clustering pattern of DEGs in BCG-WT and BCG85B-immunized mice compared to Naïve, with upregulated genes in red and downregulated in green. **(B)** Venn diagram illustrating the count and overlap of DEGs across Naïve, BCG-WT, and BCG85B groups. **(C–E)**. Volcano plots depicting the distribution of BCG-WT and BCG85B for each condition compared to the corresponding naive control. Significantly upregulated genes are highlighted in red, while downregulated genes are marked in green.

To understand how Ag85B overexpression by BCG85B impacts host gene expression, we analyzed the transcriptomic profiles of splenocytes of mice immunized with wild-type BCG (BCG-WT) and BCG85B using PolyA+ RNA-seq (**Figure 1A**). A heat map of differentially expressed genes (DEGs) (**Figure 2A**) showed strong and distinct transcriptional changes in both BCG-WT and BCG85B immunized groups compared to the unimmunized (naïve) group. Although both BCG85B and BCG-WT led to several genes being upregulated and downregulated compared to naïve cells, BCG85B had a greater effect on the gene expression profile. An intersectional Venn diagram was used to quantify the overlapping and unique sets of genes among the groups (**Figure 2B**). The analysis confirmed that a large core set of 11,723 genes was shared across all groups, supporting the idea that manipulating a single gene does not significantly alter the transcriptional profile. Despite this baseline response, both BCG-WT and BCG85B displayed a unique gene set whose expression was altered *in vivo*, with 290 and 194 exclusive DEGs, respectively. (**Figure 2B**). Furthermore, volcano plot analysis highlighted the magnitude of the host response. BCG85B-immunized mice showed a substantial number of genes significantly up- or downregulated compared to naïve cells (**Figure 2D**), a pattern also observed in BCG-WT, but less pronounced (**Figure 2C**). Notably, a direct comparison between BCG85B and BCG-WT (**Figure 2E**) identified that a total of 689 genes (226 upregulated and 463 downregulated) were differentially regulated by BCG85B, indicating its potential to regulate additional immunogenic or metabolic pathways in mice that may contribute to the host’s altered immunogenic profile.

### BCG85B induces expression of genes associated with immune pathways in mice

**Figure 3:**
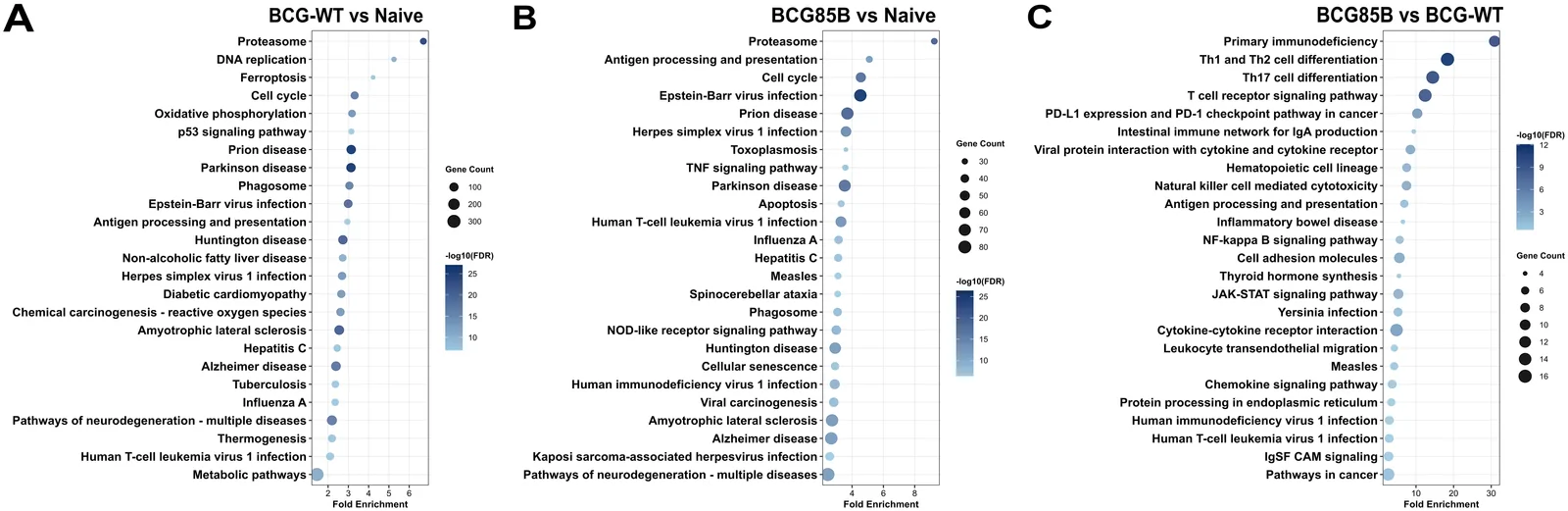
KEGG pathway enrichment analysis of upregulated genes in mice immunized with BCG-WT and BCG85B. **(A–C)** Dot plots highlighting the top-25 significantly enriched KEGG pathways associated with upregulated genes in each comparison [BCG-WT vs Naïve], [BCG85B vs Naïve], and [BCG85B vs BCG-WT], respectively. The dot size corresponds to the number of DEGs mapped to each pathway; the X-axis represents fold enrichment of each pathway, while the color gradient represents statistical significance (–log10 FDR), with warmer colors indicating stronger enrichment.

To gain mechanistic insight into how BCG85B reprograms host responses, KEGG pathway enrichment analysis was performed on the DEG set from splenocytes of immunized mice. Compared with the naïve group, splenocytes from the BCG85B-immunized group showed upregulated genes that are enriched for immunogenic pathways, including antigen processing and presentation, NOD-like receptor signaling pathway, apoptosis, and TNF signaling pathway (**Figure 3B**). These pathways indicate activation of the adaptive immune response and antigen presentation driven by Ag85B overexpression. In contrast, splenocytes from mice immunized with BCG-WT showed enrichment in pathways linked primarily to viral infection signatures, oxidative phosphorylation, neurodegenerative pathways (Parkinson’s, Alzheimer’s), and cell cycle (**Figure 3A**). While BCG-WT also activated immune pathways, the diversity and magnitude of these pathways were noticeably lower. However, direct comparison of the transcriptomes of BCG-WT and BCG85B immunized mice revealed (**Figure 3C**) that BCG85B exhibited a significantly stronger enrichment of Th1/Th2 and TH17 differentiation, NF-kappa B signaling, cytokine-cytokine receptor interactions, NK cell mediated cytotoxicity, inflammatory networks, and JAK–STAT signaling and T-cell receptor signaling, confirming that Ag85B overexpression enhances host immune response beyond the baseline induction by BCG-WT.

**Figure 4:**
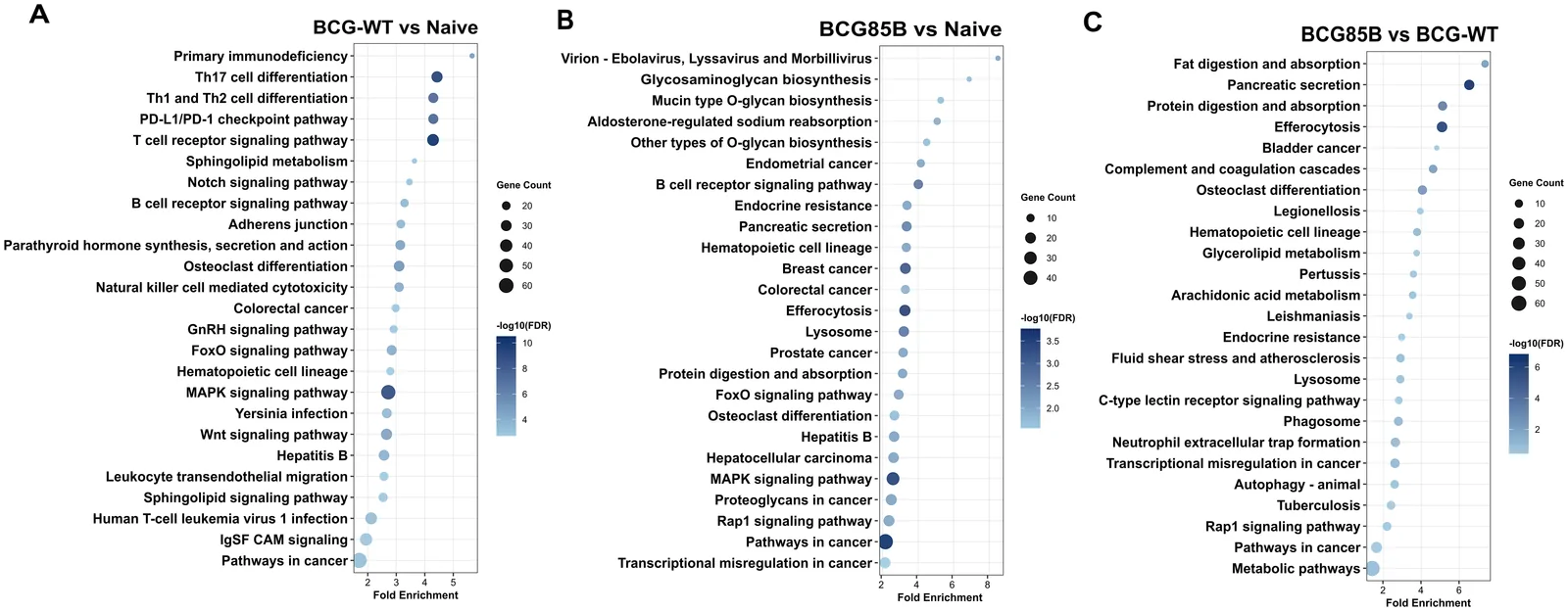
KEGG pathway enrichment analysis of down-regulated genes in mice immunized with BCG-WT and BCG85B. Dot plots illustrate that the top 25 significantly enriched KEGG pathways are associated with downregulated genes across **(A)** BCG-WT vs Naïve **(B)** BCG85B vs Naïve and **(C)** BCG85B vs BCG-WT. Each dot represents a pathway, with dot size indicating the number of genes mapped to that pathway and the color gradient reflecting statistical significance (FDR), where warmer tones denote stronger enrichment. These profiles reveal condition-specific suppression of key molecular pathways and highlight the functional consequences of transcriptional downregulation in response to the experimental treatments.

The KEGG downregulation profile further emphasized the distinct immunomodulatory behavior of BCG85B (**Figure 4**). A striking and biologically relevant divergence was seen between two strains (BCG85B and BCG-WT), as major immune pathways like T cell receptor signaling, Wnt, Notch signaling pathways, leukocyte trans endothelial migration, natural killer cell mediated cytotoxicity and T-cell differentiation pathways (Th1, Th2, Th17) which were associated with robustly upregulated genes in case of BCG85B were found to be linked with downregulated genes in BCG-WT (**Figures 4A, B**). This may explain why BCG85B elicits a more robust and protective anti-tubercular immune response. In contrast, BCG85B primarily showed downregulation of pathways involved in hormonal regulation and general physiological signaling, such as growth hormone synthesis and action, pancreatic secretion, glycosaminoglycan biosynthesis, protein/fat digestion and reabsorption, parathyroid hormone synthesis/secretion and aldosterone-regulated sodium reabsorption (**Figures 4B, C**). Taken together, the KEGG pathways associated with downregulated genes strongly suggest that BCG85B preserves immune activity, showing reduced suppression of immune pathways, whereas BCG-WT exhibits broad immune downregulation, particularly across T-cell, NK-cell, and cytokine-mediated signaling modules. These findings further strengthen BCG85B as the more immunogenic and immune-activating candidate compared with BCG-WT.

### Gene ontology analysis of mice immunized with the BCG85B strain compared to BCG

**Figure 5:**
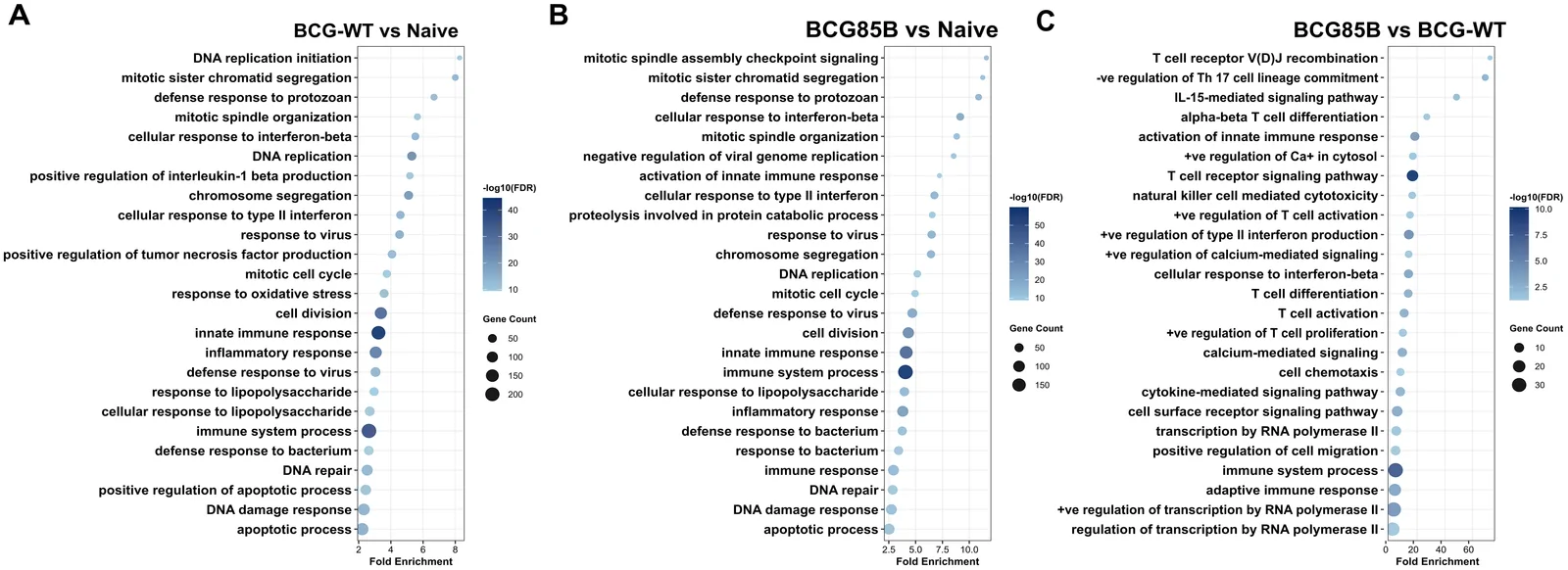
GO biological processes enrichment analysis of up-regulated genes following immunization of mice with BCG-WT and BCG85B. **(A–C)** Dot plots illustrating top-25 significantly enriched GO Biological Process terms associated with up-regulated DEGs in BCG-WT vs Naïve **(A)**, BCG85B **(B)** vs Naïve, and BCG85B vs BCG-WT **(C)** comparisons. The dot size corresponds to the number of DEGs mapped to each pathway, while the color gradient represents statistical significance (FDR), with warmer colors indicating stronger enrichment.

We analyzed GO enrichment to obtain a more detailed functional characterization of the transcriptomic changes (**Figure 5**). Splenocytes from both BCG-WT- and BCG85B-immunized mice showed the same pattern of upregulation as those from naïve mice (**Figures 5A, B**). Primarily upregulated biological processes included cellular response to interferon beta, response to virus, cell division, DNA replication, and apoptosis. However, activation of innate and inflammatory immune responses is among the immune-related processes linked to only BCG85B-induced genes. A direct comparison between the BCG85B and BCG-WT groups further confirmed the better immune-stimulatory profile of BCG85B (**Figure 5C**). Most of the top biological processes linked to upregulated genes exclusive to the BCG85B group were associated with immune defense and response. Processes such as activation of immune response, α-β T cell activation and differentiation, T-cell production and differentiation, IL-15 mediated signaling, cytokine mediated signaling pathway, positive regulation of immune response, response to and production of interferon-beta, T-cell differentiation and activation, +ve regulation of T cell proliferation, +ve regulation to interferon II production, and activation of immune response were upregulated by BCG85B, further indicating that Ag85B overexpression enhances both innate and adaptive immune activation.

**Figure 6:**
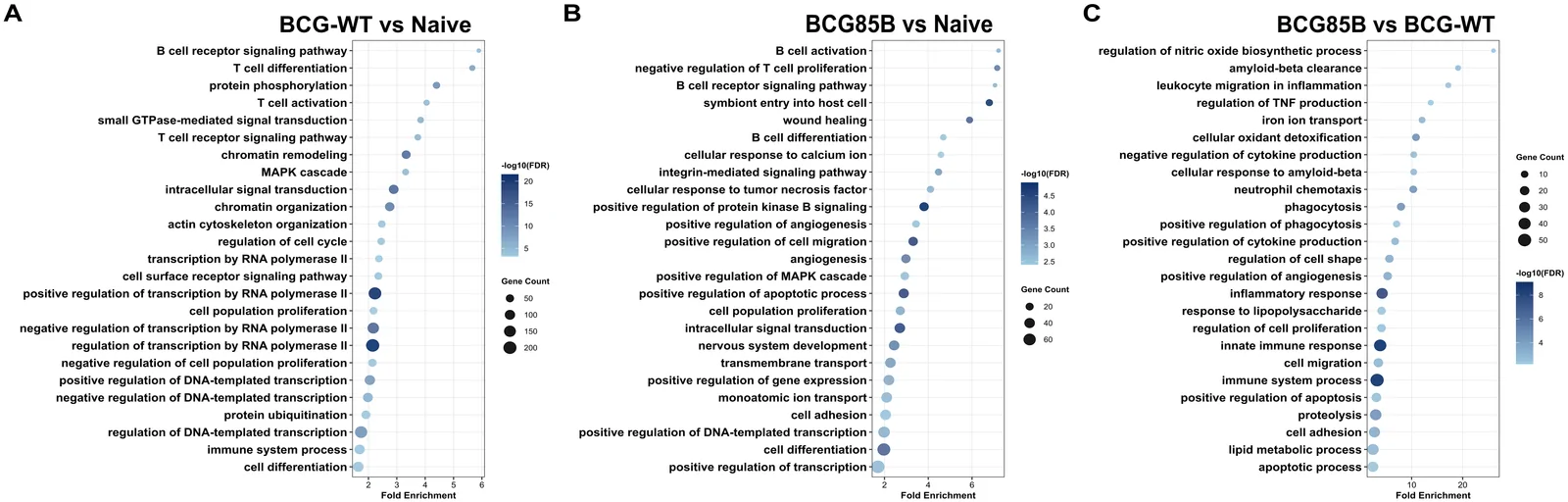
GO biological processes analysis of down-regulated genes following immunization of mice with BCG-WT and BCG85B. **(A–C)** Dot plots showing the top 25 significantly enriched GO Biological Process terms associated with down-regulated DEGs in BCG-WT vs Naïve **(A)**, BCG85B vs Naïve **(B)**, and BCG85B vs BCG-WT **(C)**. The dot size indicates the number of genes involved in each pathway, and dot color denotes statistical significance (FDR), with warmer colors indicating stronger enrichment.

Upon analyzing the enrichment of biological processes among downregulated genes, we observed a trend similar to that observed in KEGG pathways. Compared with the naïve group, the BCG-WT immunized group showed an association between downregulated genes and immune-related processes, including T-cell-specific processes such as T-cell activation, T-cell differentiation, and T-cell receptor signaling, as well as the MAPK cascade (**Figure 6A**). Notably, these critical immune processes were not associated with downregulated genes in the BCG85B-immunized group, suggesting that the recombinant strain retains the ability to induce transcription and activate T cells (**Figure 6B**). Furthermore, upon direct comparison of transcriptomics between BCG85B and BCG-WT immunized cells (**Figure 6C**), there was a selective downregulation of genes linked to cell regulatory processes such as, cellular oxidant detoxification, cellular response to amyloid-beta, cell migration, cell adhesion, regulation of cell proliferation, and regulation of nitric oxide biosynthesis, suggesting a shift towards a more energy-efficient basal state.

### Comparative analysis of transcriptional factors involved in the regulation of DEGs for mice immunized with BCG85B and BCG-WT

**Figure 7:**
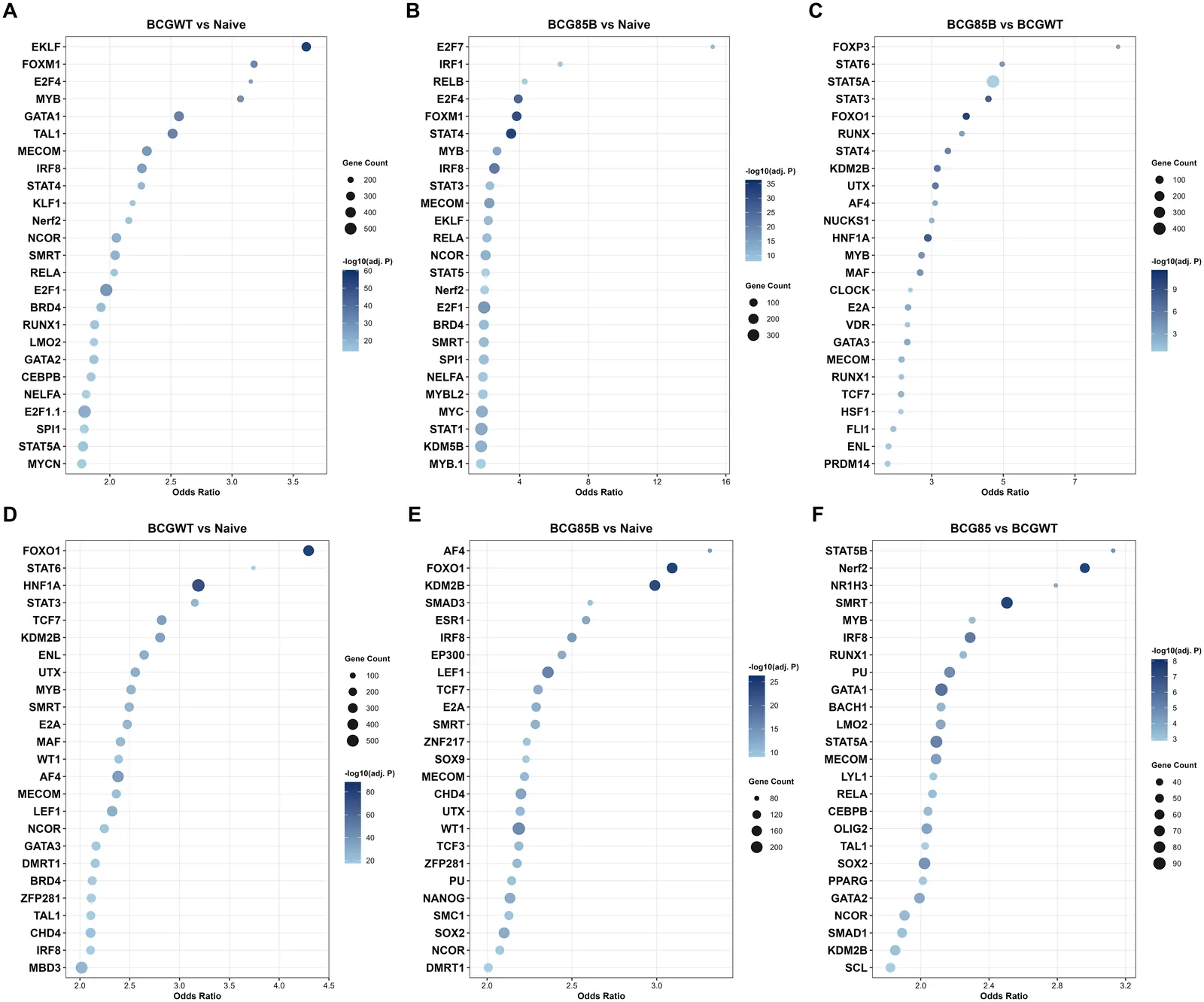
TFs that target differentially regulated genes across pairwise comparisons of mice immunized with BCG-WT and BCG85B. **(A–C)**. Represent the top TFs linked to upregulated genes identified in the comparisons between BCG-WT vs Naïve **(A)**, BCG85B vs Naïve **(B)**, and BCG85B vs BCG-WT **(C)**, respectively. **(D–F)** Depict the top TFs linked to downregulated genes for the corresponding comparisons: BCG-WT vs Naïve **(D)**, BCG85B vs naïve **(E)**, and BCG85B vs BCG-WT **(F)**. Transcription factors were selected based on differential expression analysis, and the regulation status (up- or down-regulated) reflects relative expression changes between the compared groups.

We further investigated whether the targeted genes were controlled by the same or different transcription factors (TFs). We observed that the overall TF network was similar in both the BCG-WT and BCG85B-immunized groups compared with the naïve group (**Figures 7A, B, D, E**). When comparing the BCG85B group with the BCG-WT group (**Figure 7C**), we observed that several TFs involved in T-cell differentiation and effector programming, such as STAT3, STAT6, STAT4, RUNX, GATA3, FOXO1, MYB, TCF7, and MAF, were associated with upregulated genes in the BCG85B group. These factors are known to induce the formation of Th1, Th17, Th3, and memory T cells (42) and T- cell activation (43). Further, pathways such as UTX, KDM2B, E2A, and AF4, which are associated with transcriptional accessibility and chromatin remodeling (44) and support rapid immune gene expression (45). Factors such as VDR and NUCKS1, both linked to antimicrobial response and macrophage function, were also linked to BCG85B-induced genes. In contrast, TFs associated with downregulated genes in BCG85B relative to BCG-WT (**Figure 7F**) are mainly involved in hematopoiesis and erythroid lineage differentiation, including GATA1, MECOM, LYL1, and LAMO2. Additionally, regulators of lipid metabolism, including NR1H3 and BACH1, appear to target downregulated genes. Finally, TFs such as RELA, which are involved in general transcription and signaling, target downregulated genes.

### BCG85B and BCG-WT regulate distinct signaling networks

**Figure 8:**
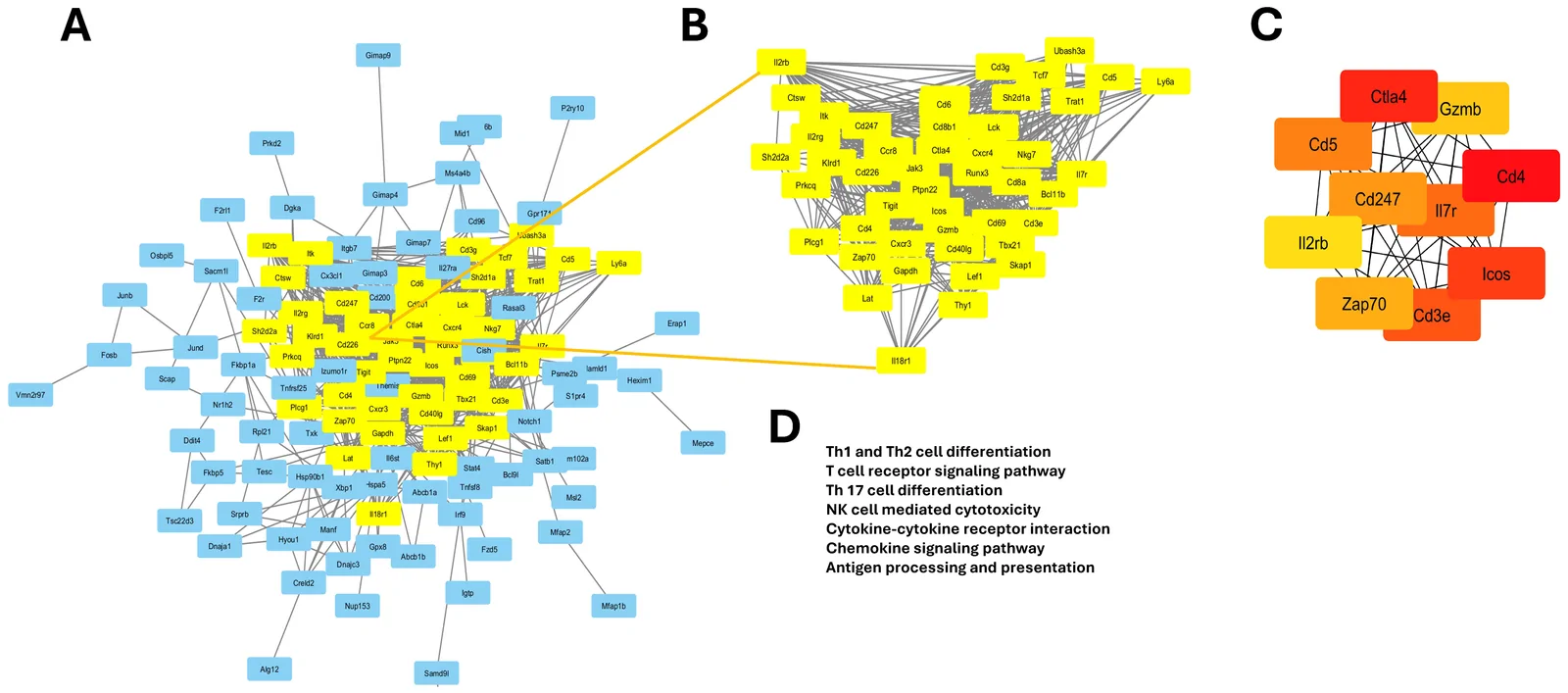
Protein–protein interaction (PPI) and functional enrichment analysis of DEGs between BCG85B and BCG-WT. STRING-based PPI and functional enrichment analyses were performed on DEGs identified between BCG85B and BCG-WT. **(A)** Clusters derived from the STRING PPI network were visualized in Cytoscape. Module detection was performed using the MCODE plugin to identify highly interconnected functional clusters (in yellow). **(B)** Subnetwork representing the key module identified from the PPI network in **(A)** using the MCODE algorithm. **(C)** Identification of hub genes from the top MCODE-derived cluster using the CytoHubba plugin (MCC method), representing key regulatory proteins within the PPI network. **(D)** Functional enrichment analysis of genes within the top MCODE-identified cluster was performed using DAVID, highlighting the immune-related enriched KEGG pathways.

**Figure 9:**
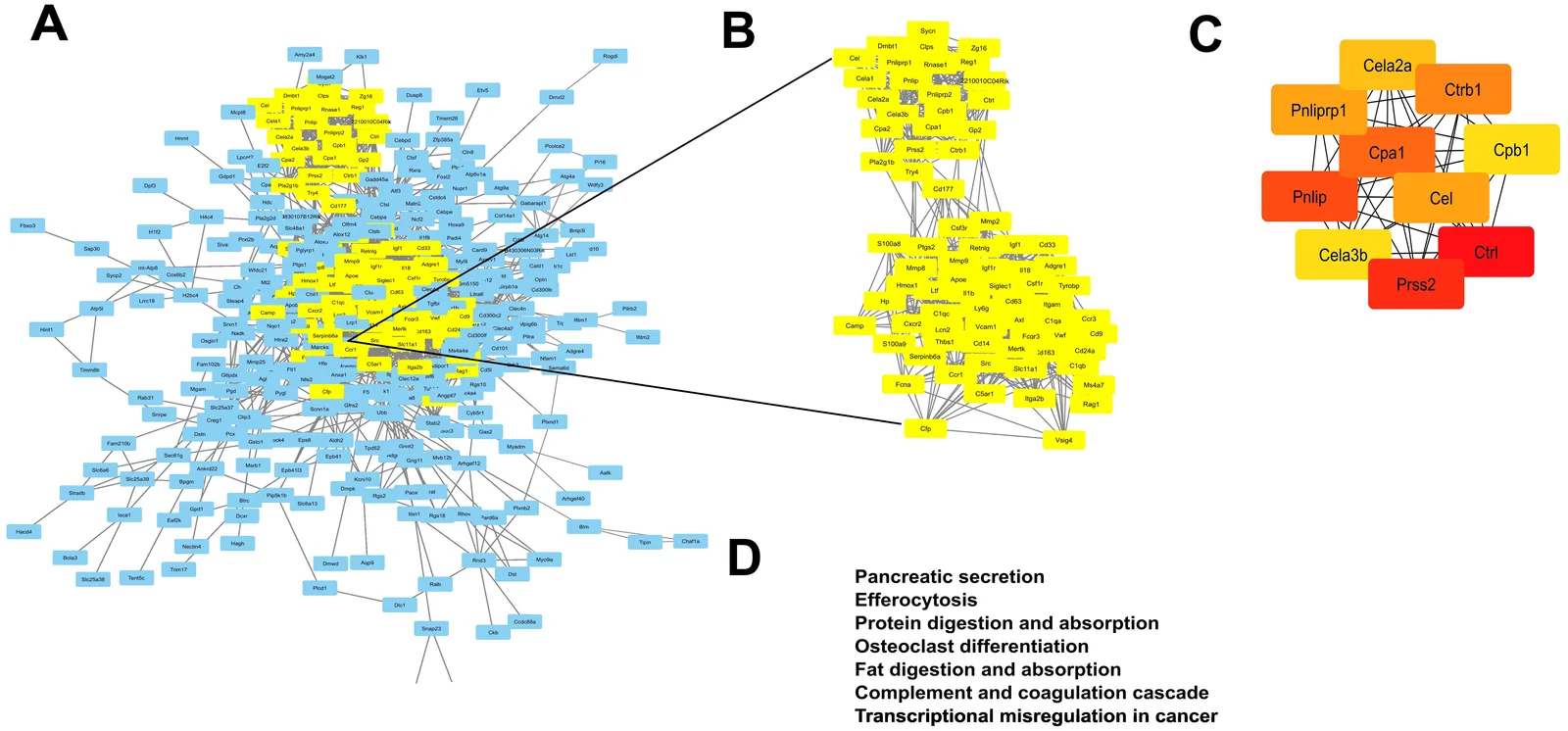
PPI and functional enrichment analysis of DEGs between BCG85B and BCG-WT. STRING-based PPI and functional enrichment analyses were performed on DEGs identified between BCG85B and BCG-WT. **(A)** Clusters derived from the STRING PPI network were visualized in Cytoscape. Module detection was performed using the MCODE plugin to identify highly interconnected functional clusters (in yellow). **(B)** Subnetwork representing the key module identified from the PPI network in **(A)** using the MCODE algorithm. **(C)** Identification of hub genes from the top MCODE-derived cluster using the CytoHubba a plugin, representing key regulatory proteins within the PPI network. **(D)** Functional enrichment analysis of genes within the top MCODE-identified cluster was performed using DAVID, highlighting the top 7 enriched KEGG pathways.

Furthermore, to explore functional interactions among DEGs (upregulated and downregulated) between BCG85B and BCG-WT, PPI networks were constructed using the STRING database (**Figures 8**, **9**). The PPI networks were visualized in Cytoscape (**Figures 8A**, **9A**), and module detection was performed using the MCODE plugin to identify highly interconnected clusters. The most significant cluster, with the highest score, was extracted for further analysis (**Figures 8B**, **9B**). HUB gene analysis of this cluster identified *CD5*, *CD3E*, *ZAP70*, *IL2RB*, *CD247*, *CTLA4*, *CD4*, *GZMB*, *IL7R*, and *ICOS* as the top 10 most upregulated genes (**Figure 8C**). All these genes are known to have immunogenic functions (46–48). Furthermore, the selected cluster was uploaded onto DAVID to check for significantly enriched KEGG pathways (**Figure 8D**). KEGG pathways highlighted T-cell receptor signaling, Th1/Th2 differentiation, cytokine-cytokine receptor signaling, and antigen presentation amongst the top enriched (**Figure 8D**). Together, this implies that BCG85B induces a strong immune signaling pathway, pointing towards an enhanced immunomodulatory state as compared to BCG.

In contrast to upregulated HUB genes, *Cpa1*, *Cela2a*, *Prss2*, *Pnliprp1*, *Cpb1*, *Ctrl*, *Cela3b*, *Pnlip*, *Cel*, and *Cela3b* were amongst the top downregulated genes based on the HUB gene analysis of the enriched PPI network (**Figure 9C**). These genes are predominantly associated with pancreatic exocrine function and protein and lipid digestion. Furthermore, the KEGG pathway analysis confirmed the same finding: the top enriched downregulated pathways in the network included pancreatic secretion, fat/protein digestion and absorption, efferocytosis, and osteoclast differentiation (**Figure 9D**).

### BCG85B enhances Ag presentation and antigen-specific recall cytokine responses along with reduced mycobacterial burden

**Figure 10:**
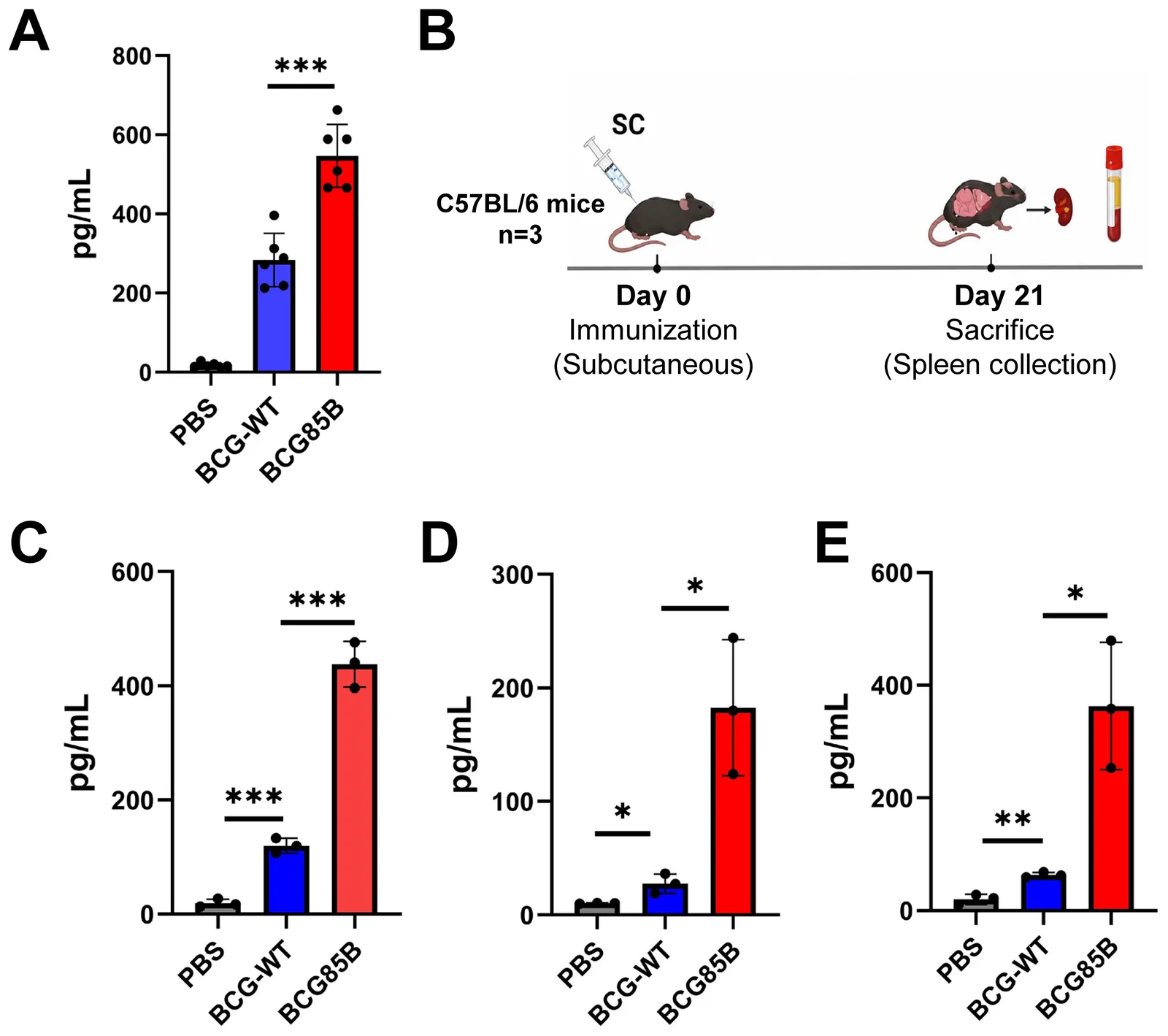
Enhanced antigen presentation and cytokine responses following immunization with BCG85B. **(A)** *In vitro* antigen presentation assay using bone marrow-derived macrophages (BMDMs). BMDMs isolated from C57BL/6 mice were infected or treated with PBS (control), BCG, or BCG85B at a multiplicity of infection (MOI) of 5 for 4 h. Following removal of extracellular bacteria, cells were co-cultured with the BB7 T-cell hybridoma for an additional 24 h, after which antigen presentation was assessed by measuring IL-2 secretion in the culture supernatants by ELISA. **(B)** Schematic representation of the experimental design for immunization and cytokine analysis. C57BL/6 mice were immunized subcutaneously with BCG and BCG85B on Day 0 and sacrificed on Day 21. Splenocytes were isolated and stimulated *ex vivo* for cytokine analysis. **(C–E)** Cytokine responses of splenocytes isolated from immunized mice at Day 30 following *ex vivo* stimulation. Concentrations of **(C)** IFN-γ, **(D)** IL-2, and **(E)** TNF-α in culture supernatants were quantified by ELISA. Data are presented as mean ± SEM. Statistical significance was determined using one-way ANOVA followed by Tukey’s multiple-comparison test. *P* < 0.05 was considered statistically significant.Statistical significance was defined as follows: ns, not significant (P ≥ 0.05); * P < 0.05; ** P < 0.01; *** P < 0.001; **** P < 0.0001.

To functionally confirm the improved immunogenicity suggested by transcriptomic profiling, we first evaluated the antigen-presenting ability of bone marrow-derived macrophages (BMDMs) immunized with BCG85B or BCG-WT. Compared to BCG-WT, BCG85B-infected BMDMs induced significantly higher levels of IL-2 production via BB7 cells (**Figure 10A**), suggesting elevated processing and presentation of Ag85B-derived antigenic peptides through the MHC-II pathway. Furthermore, to determine if the increased antigen presentation translated into improved adaptive immune response, ex vivo cytokine analysis was performed using murine splenocytes (**Figure 10B**). Splenocytes from BCG85B-immunized mice produced significantly higher levels of IL-2, TNF-α, and IFN-γ than those from BCG-WT-immunized mice (**Figures 10C–E**). The increased production of these cytokines indicates that Ag85B overexpression promotes a robust antigen-specific recall response, marked by elevated secretion of Th1-associated cytokines and functional immune activation. This is consistent with the transcriptomic studies demonstrating enhanced cytokine signaling and T-cell receptor pathways. To further evaluate if immune enhancements translated to *in vivo* control of bacterial growth, a distinct cohort of immunized C57BL/6 mice was challenged with aerosolized Mtb (**Supplementary Figure 1A**). BCG85B immunization resulted in a reduced bacterial load compared with BCG-WT-immunized mice in both the lungs and the spleen (**Supplementary Figures 1B, C**), suggesting enhanced control of mycobacterial replication.

## Discussion

Ag85B, a secreted antigenic protein of Mtb, induces robust Th1-based CD4+ and CD8+ immune responses (31, 49, 50) and is a preferred antigen for TB vaccine development across multiple platforms. Recombinant BCG (rBCG) strains engineered to overexpress Ag85B alone (30, 31, 51) or in combination with other antigens (19) have demonstrated enhanced protection against virulent Mtb, resulting in superior long-term lung protection and increased IFN-γ responses compared to wild-type BCG (BCG-WT) (52–54). In the present study, BCG85B, which overexpresses Ag85B, induced a distinct set of DEGs in splenocytes compared to BCG-WT (**Figure 2**). Heatmap analysis comparing the DEGs induced by these strains with those of naïve controls revealed that BCG85B induced a unique set of DEGs, potentially contributing to the well-documented immunodominance and antigen-presenting capabilities of Ag85B. Additionally, volcano plot analysis indicated that cells from mice immunized with BCG85B overexpressed 689 genes relative to cells from mice immunized with BCG-WT, of which 226 were upregulated and 463 were downregulated. This expression pattern is consistent with previous findings that extensive DEG profiles are often associated with significant functional changes in bacterial systems (55).

A broad spectrum of KEGG pathways was enriched in BCG85B-immunized mice compared with those immunized with BCG-WT. Most of these pathways are associated with immunological functions, including Th1, Th2, and Th17 cell differentiation, T-cell receptor signaling, NF-kappa B signaling, JAK-STAT signaling, antigen processing and presentation, NK cell-mediated cytotoxicity, Notch signaling, and cytokine-cytokine receptor interaction, which collectively indicate robust immune system activation (56, 57). The enrichment of these pathways may contribute to the enhanced immune protection previously observed in Ag85B-overexpressing BCG strains compared with their wild-type counterparts. These findings are consistent with prior studies demonstrating that cytokine signaling dynamics, increased antigen availability, and enhanced T-cell priming are linked to Ag85B immunogenicity (58–60). Compared to BCG-WT, the elevated enrichment of the JAK-STAT pathway and cytokine-cytokine receptor interaction, which are hallmarks of cytokine-driven immune responses essential for interferon signaling, macrophage activation, and T-cell-dependent differentiation, may underlie the superior protection conferred by Ag85B. O’Garra et al. (61) reported that CD4+ T cells and the cytokines IL-2, IFN-γ, and TNF are critical for enhanced protection against Mtb. The present study also demonstrated greater enrichment of Th1, and Th17-related pathways in the splenocytes of mice immunized with BCG-85B. Deng et al. (58) similarly observed that the recombinant Ag85B-ESAT-6 strain elicited a stronger and more sustained Th1-type cellular immune response than BCG. Pathways associated with cell-mediated cytotoxicity, such as NK cell activation, that facilitate the elimination of infected host cells were also enriched in BCG85B-immunized mice. These results support the conclusion that Ag85B overexpression may promote coordinated activation of both helper (CD4+-mediated) and effector (CD8+-mediated) T-cell-associated immunity in the host cells.

In contrast, KEGG pathways enriched for downregulated genes were primarily associated with metabolism, protein digestion and absorption, fat digestion and absorption, arachidonic acid metabolism, glycerolipid metabolism, and starch and sucrose metabolism in splenocytes from BCG85B-immunized mice. Additionally, signaling pathways such as C-type lectin receptor signaling and Rap1 signaling were also linked to downregulated genes. This reduction in metabolic and signaling network activity suggests that Ag85B may prompt the host to prioritize antigenic stimulation, resulting in a heightened immunogenic response at the expense of metabolic functions. Sun et al. (61), previously demonstrated that metabolic reprogramming occurs within the host during emergencies, favoring the immune response over basal metabolic functions. Collectively, these findings indicate that Ag85B overexpression not only activates immune-related pathways but also balances metabolic networks to prioritize immune responses while restricting less essential or potentially harmful activities.

Both BCG85B and BCG-WT were found to enrich distinct biological processes compared with the naïve group. BCG85B-induced genes are linked to numerous immune-related biological processes, including T cell activation, adaptive immune response, T cell differentiation, regulation of T cell activation, T cell proliferation, and immune response activation, relative to BCG. Notably, 9 of the top 25 upregulated enrichment processes were associated with T-cell differentiation, activation, and functioning, underscoring the central role of T cells in mediating the immune response induced by Ag85B. Previous studies have consistently shown that the immunogenic effect of Ag85B is primarily driven by antigen-specific T-cell responses (49, 62, 63). In contrast to BCG-WT, BCG85B downregulated biological processes mainly related to cellular transport and migration, such as cell migration, iron ion transport, neutrophil chemotaxis, cell chemotaxis, myeloid leucocyte migration, leukocyte migration, granulocyte migration and chemotaxis, and cellular entry. This pattern suggests that BCG85B may suppress certain homeostatic and motility programs to prioritize immune function. Previous studies have also demonstrated that immune-related genes and pathways are upregulated during Mtb infection compared to basal metabolism and migration-related functions (64, 65).

All TFs that potentially target upregulated genes were primarily involved in regulating immune-related responses, including JAK-STAT signaling, T-cell differentiation, cytokine signaling, and immune homeostasis. Upregulated STAT family TFs (STAT4, STAT5A, STAT3, and STAT6) play a major role in JAK-STAT signaling and IL-2/4- and IL-12-associated functions (66). Enrichment of TFs such as TCF1/TCF7, GATA3, RUNX1, and MYB is known to play a vital role in T-cell differentiation and maintenance (67, 68). TFs such as MAF, VDR, and FOXP3 indicate activation of anti-inflammatory pathways that appear to target upregulated genes. FOXP3 is a known regulator of CD4+ T cells and is essential for memory formation, while MAF2 enhances IL-10 production and regulatory phenotypes during immune activation (69).

**Figure 11:**
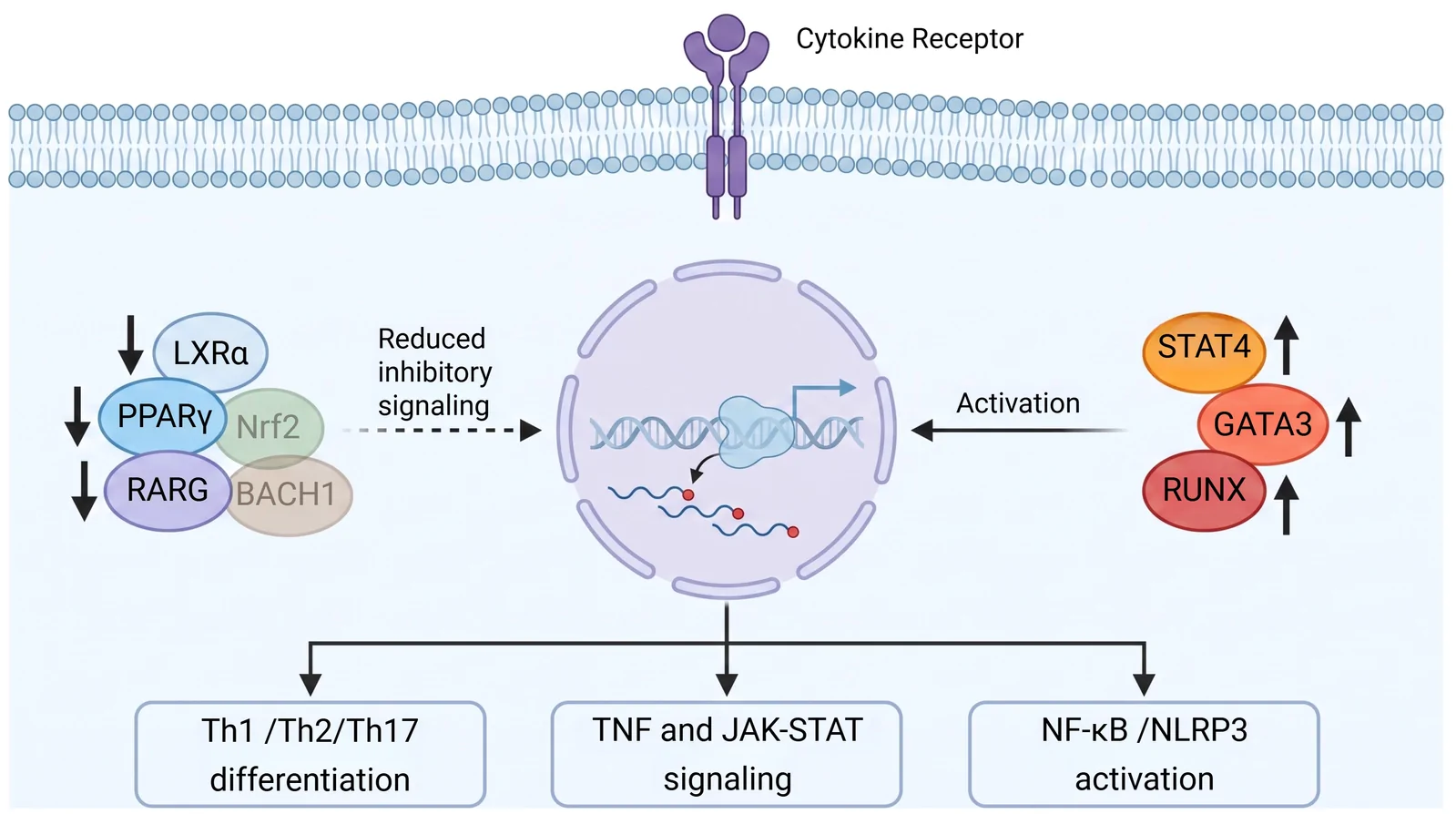
Proposed mechanistic model linking transcriptional loss of lipid-sensing and redox-regulatory nuclear receptors to enhanced effector immune programs in BCG85B-immunized splenocytes. Downregulation of nuclear receptors like PPARG, NR1H3 (LXRα), and RARG that normally restrain Th17/Th1 differentiation and promote Foxp3^+^ Treg induction was accompanied by lower expression of lipid metabolism-associated KEGG pathways (fat digestion and absorption, glycerolipid metabolism, arachidonic acid metabolism), suggesting lower availability of their inhibitory lipid ligands. At the same time, redox-sensitive repressors Nrf2 and BACH1, which restrain NF-κB/NLRP3 inflammasome-driven cytokine transcription, were also downregulated. This aligns with the enrichment of immune pathways, such as NOD-like receptor and TNF signaling pathways, in BCG85B-immunized mice. Together, these changes suggest reduced inhibitory control over immune gene regulation, lowering the threshold for STAT4-, GATA3-, and RUNX-driven effector programs and contributing to the enhanced Th1/Th2/Th17, JAK-STAT, and cytokine-cytokine receptor signatures observed in BCG85B relative to BCG-WT. Image was prepared using Biorender.

In contrast, TFs that appear to target the most downregulated genes were predominantly associated with cellular development and stress response, chromatin organization, and metabolic homeostasis. TFs such as TAL1, GATA1/2, MYB, KDM2B, SOX2, and MECOM are primarily associated with hematopoietic development, epigenetic stability, and lineage maintenance. Many TFs linked to downregulated genes were also associated with nuclear receptors, including NR1H3 (LXRα), PPARG (PPARγ), and RARG (retinoic acid receptor γ), which are primarily involved in differentiation and lipid metabolism and are known to limit Th cell response. LXRα can suppress Th17 differentiation by inducing Srebp-1, which interferes with aryl hydrocarbon receptor-driven IL-17 transcription, and reciprocally promotes Foxp3 regulatory T cell induction upon activation (70). Similarly, PPARγ is known to selectively suppress Th17 differentiation by preventing the removal of corepressor protein from the Rorc gene promoter (71). Retinoic acid signaling through RAR supports Foxp3 induction via TGF-β/Smad3 signaling and inhibits IL-6/IL-23 receptor expression, which is essential for Th17 signaling (72). The simultaneous downregulation of these nuclear receptor target genes in BCG85B-immunized splenocytes suggests a coordinated loss of this anti-inflammatory regulatory network. This is consistent with enrichment of lipid metabolism-related KEGG pathways among the down-regulated, including fat digestion and absorption, glycerol metabolism, and arachidonic acid metabolism. Together, these findings suggest that reduced lipid metabolism may lead to fewer natural ligands for these receptors, thereby weakening their suppressive effect and allowing stronger T cell activation. This interpretation is further supported by parallel downregulation of redox-sensitive repressors Nrf2 and BACH1, both of which suppress NF-κB and NLRP3 inflammasome-dependent cytokine transcription (73, 74). Their decreased expression may contribute to the concurrent enrichment of NOD-like receptor and TNF signaling pathways observed in BCG85B-immunized mice. Together, these findings support a model in which Ag85B overexpression is associated with coordinated downregulation of lipid sensing and redox-regulatory transcriptional network, thereby lowering the threshold for STAT4-, GATA3-, and RUNX-driven effector transcriptional regulation programs and leading to a stronger Th1/Th2/Th17 cytokine receptor signature that distinguishes BCG85B from BCG-WT (**Figure 11**). We acknowledge that this model is correlational and based on transcriptional enrichment; direct validation, such as pharmacological modulation of LXR/PRAPγ, will be needed to confirm this mechanism in future studies. Some TFs, such as IRF8, RELA, STAT5A/5B, and SPI1 (PU.1), that may target downregulated genes, are involved in immune regulation but are inherently multifunctional, contributing to cellular survival, homeostasis, and differentiation.

The PPI network and enrichment HUB gene analyses revealed two distinct expression patterns between BCG85B and BCG-WT groups, highlighting a coordinated shift in the cellular functional state. Upregulated genes, *CD3E*, *CD5*, *ZAP70*, *IL7R*, *GZMB*, *ICOS*, *CTLA4*, *IL2RB*, are mainly associated with T cell receptor (TCR) signaling, cytokine-mediated signaling, cytotoxic effector functions, and immune checkpoints (63, 75–77). CD3E, ZAP70, and CD247 form the core signaling complex for TCR signaling, which is central for antigen recognition and immune activation (76). Activation of these genes (pathways) leads to T cell differentiation, cytokine production, and cytotoxic activity. Immune checkpoints, such as CTLA-4, and receptors, such as ICOS, are essential for regulating T cell responses and balancing immune modulation (78, 79). Furthermore, IL2RB, IL7R, and GZMB were upregulated, resulting in enhanced cytokine production and cytotoxic lymphocyte production, both of which are associated with immune activation (80, 81). In contrast, the downregulated HUB genes-*Cela2a*, *Prss2*, *Cpb1*, *Pnlip*, *Cpa1*, and *Cel*-are predominantly associated with pancreatic exocrine function and digestive enzyme activity (82). This inverse relationship between immune activation and metabolic/digestive gene expression reflects the biological phenomenon known as functional reprogramming, which posits that during immune activation, cellular resources are diverted from homeostasis toward immune defense mechanisms (61, 83–85). Upon analyzing KEGG pathways, it was evident that upregulated gene sets formed dense clusters in STRING immune-related networks, confirming that Ag85B-driven activation induces robust immune programs. In contrast, downregulated networks were primarily associated with metabolic functions. Therefore, the STRING-based PPI analysis validated the DEG-based pathway enrichment analysis and demonstrated that the observed transcriptional changes reflect coordinated and biologically meaningful programs rather than isolated gene expression events.

The transcriptomic changes identified following BCG85B immunization were further reflected at the functional level through improved antigen presentation, antigen-specific recall responses, and enhanced control of Mtb infection. Among the substantially enriched KEGG pathways, antigen processing and presentation emerged as one of the most notable immune pathways, indicating that Ag85B strengthens the link between innate antigen-presenting cells and adaptive T-cell activation. Consistent with this observation, BCG85B-infected BMDMs exhibited significantly enhanced activation of the Ag85B-specific BB7 T-cell hybridoma, as indicated by increased IL-2 secretion. Effective antigen processing and presentation are critical to vaccine-induced cellular immunity, directly impacting T-cell priming, clonal expansion, and subsequent memory formation. Previous studies similarly showed that Ag85B-overexpressing rBCG induces enhanced antigen-specific T-cell activation compared to BCG-WT, consistent with the notion that elevated antigen availability improves adaptive immune priming (3, 30, 73, 74, 86).

The augmented antigen presentation observed *in vitro* was associated with a significantly higher antigen-specific recall response following immunization. Upon Mtb lysate stimulation, splenocytes from BCG85B-immunized mice produced significantly higher levels of TNF-α, IL-2, and IFN-γ than those from BCG-WT-immunized mice, indicating a more robust Th1-oriented memory response, as evidenced by the most upregulated KEGG pathways. Rather than representing independent findings, these datasets collectively illustrate a coordinated immune program in which elevated antigen presentation drives enhanced T-cell activation, resulting in increased effector cytokine production. IFN-γ remains crucial for macrophage activation and intracellular mycobacterial control (87, 88), whereas TNF-α functions in combination with IFN-γ to promote formation of granuloma and bacterial restriction (89, 90), and IL-2 supports antigen-derived T-cell proliferation, growth, and memory conservation (91, 92). A similar increase of Th1-associated cytokines has regularly been observed following vaccination with the Ag85B-expressing BCG strain, further strengthening the biological relevance of our observation (3, 30, 93–96). Together, these outcomes deliver functional evidence that the immune pathways noted by transcriptomic analysis translate into improved antigen-specific cellular immune responses.

Remarkably, these synchronized immunological responses were further reflected in the *in vivo* challenge model, where BCG85B-immunized mice showed reduced bacterial burdens in both lungs and spleens following aerosol Mtb infection when compared to BCG-WT-infected mice. These findings suggest that the elevated immune activation detected at the transcriptomic level is associated with improved confinement of Mtb growth *in vivo*. Prior studies examining Ag85B-overexpressing rBCG exhibited similar results, with stronger antigen-specific cellular immunity and lower bacterial loads in the lungs and spleen following Mtb infection compared with conventional BCG-WT-immunized mice (30, 86, 97). Additionally, the combined upregulation of genes associated with immune pathways and suppression of metabolic pathways, as observed in our RNA-seq dataset, is coherent with the concept of immune metabolic reprogramming during T-cell differentiation and macrophage activation, where immune activation is associated with remodeling of cellular metabolic pathways to sustain antimicrobial immune functions (84, 98, 99).

Collectively, the convergence of transcriptomic, functional, and *in vivo* findings in this research indicates that BCG85B induces system-level immune reprogramming, characterized by highly interconnected regulatory and signaling networks. This coordinated transcriptional remodeling provides mechanistic insight into how Ag85B overexpression modulates host immunity beyond the activation of isolated pathways.

This study provides an extensive transcriptomic and functional examination of immune responses induced by BCG85B vaccination. The analysis performed at a defined time point measured the adaptive immune state and reflected population-level transcriptional changes. Further studies integrating time-course-based sampling and single-cell transcriptomics strategies will further refine the temporal and cellular details of these responses. In addition, long-term protective immunity and translational studies in human immune systems will be beneficial to further determine the significance of these findings for TB vaccine development.

## Materials and methods

### Bacterial strains and culture conditions

The strains utilized in this study, BCG85B and BCG-WT (Pasteur), were cultured at 37 °C in either 7H10 agar (BD Difco) or 7H9 broth supplemented with 0.2% glycerol, 0.05% Tween 80, and 10% ADC enrichment. BCG85B is an Ag85B-overexpressing strain, the construction of which has been previously described (100).

### Animals and ethics

C57BL/6J mice, aged four to six weeks, were obtained from Jackson Laboratories, Bar Harbor, ME. Upon arrival, the animals were housed at the Laboratory Animal Resource Center (LARC) at Texas Tech University Health Sciences Center El Paso. Mice were provided with unrestricted access to standard chow and water and were allowed free movement within their cages. The animal study was approved by the Institutional Animal Care and Use Committee (IACUC) of Texas Tech University Health Sciences Center El Paso, which approved the animal protocol for this study. The study was conducted in accordance with the local legislation and institutional requirements. At the experimental endpoint, mice were humanely euthanized by overdose of isoflurane administered by inhalation (5% or greater). Adequate depth of anesthesia was confirmed by the absence of the eye blink reflex, loss of the pedal withdrawal (toe pinch test), and cessation of respiration. A cervical dislocation was used as a secondary method to verify death, in accordance with the approved animal care protocol.

### Infection of mice with bacterial strains

Bacterial strains BCG-WT and BCG85B were cultured in 7H9/ADC broth, and growth was monitored by measuring optical density at periodic intervals. When cultures reached an OD600 of approximately 1.0, bacteria were collected by centrifugation, washed, and resuspended in phosphate-buffered saline (PBS). To ensure a uniform suspension and eliminate clumps, the bacterial solution was repeatedly passed through a 23-gauge syringe. Each experimental group (Naïve, BCG85B, BCG-WT) consisted of three mice (n=3), which were immunized subcutaneously with 1 × 10^6 cells in 100 µl of PBS of the corresponding bacterial culture (BCG-WT or BCG85B). The Naïve group received an equivalent volume of PBS.

### Tissue collection, RNA isolation, RNA sequencing, and data analysis

At 21 days post-immunization, mice were euthanized, and spleens were aseptically collected in 5 mL of RPMI medium. Spleen tissues were homogenized, and total RNA was isolated using a standard RNA extraction protocol with the EZ-10 DNAaway RNA Mini-Preps Kit (Bio Basic, Canada). RNA quantity and quality were analyzed using a Nanodrop spectrophotometer (Thermo Scientific, USA) and TapeStation system (Agilent Technologies), respectively. Library preparation and sequencing were conducted by Novogene Corporation Inc. (Sacramento, USA). Raw data were processed and quality checked by Novogene, and the cleaned data were subsequently provided to the research team. Briefly, RNA sequencing libraries were prepared and sequenced on Illumina NovaSeq X Plus platform using sequencing by synthesis technology. Raw reads were processed to remove adapter sequences, and clean reads were aligned to the *Mus musculus* genome (mm39) using HISAT2. Gene expression levels were quantified from mapped reads and normalized to fragments per kilobase of transcript per million mapped reads (FPKM). Downstream analysis was performed to compare gene expression profiles among the strains. DEGs were identified using the DESeq2 package in R. Genes with p-value ≤ 0.05 and absolute log2 fold change (|log2FC|) ≥ 0 were considered significantly differentially expressed (Data Sheet 1).

Functional enrichment analysis of DEGs was performed using the DAVID bioinformatics platform. KEGG (Data Sheet 2,3) and GO (Data Sheet 4,5) pathway analyses were performed to identify significantly enriched biological processes and pathways associated with the DEGs. The top 25 enriched pathways (by significance) were visualized in R as dot plots using ggplot2. To identify key regulatory elements associated with transcriptional changes, the DEG lists from each group were subjected to transcription factor (TF) enrichment analysis. Binding motifs and regulatory networks of TFs were examined using a ChIP-based database, and the top TFs were visualized on dot plots generated with ggplot2 in R (Data Sheet 6,7). Further, DEGs (upregulated and downregulated) for BCG85B vs BCG-WT were uploaded to the STRING database (Version 12.0) to construct PPI-networks. The generated networks were imported to Cytoscape (version 3.9.1) for visualization and downstream analysis. The Molecular Complex Detection (MCODE) plugin was applied to identify the highly interconnected regions within the network. Hub genes were then identified in the top MCODE-identified cluster using CytoHubba a plugin in Cytoscape. Furthermore, functional annotation of genes within the MCODE cluster was performed using DAVID. KEGG enrichment analysis was conducted to identify significantly overexpressed biological pathways.

### Isolation of bone marrow-derived macrophages

Bone marrow-derived macrophages (BMDMs) were isolated as previously described (101). Briefly, C57BL/6 mice were euthanized, and femurs were excised and cleaned of skin and muscle under aseptic conditions. Bone marrow containing hematopoietic stem cells was flushed from the bones using 5 mL of DMEM culture medium delivered through a syringe. The resulting cell suspensions were centrifuged, and cell numbers were determined. Cells were differentiated into macrophages by culturing in DMEM containing FBS (10%, v/v) and M-CSF (10 ng/mL) at 37 °C in 5% CO_2_ in Petri dishes.

### *In vitro* antigen presentation assay

To determine the processing and presentation of Mtb antigen by BCG-infected cells *in vitro*, BMDMs (1 × 10^5 cells) were plated in a 24-well plate and stimulated with recombinant mouse INF-γ for 12 h at 37 °C in 5% CO_2_. The monolayers were later washed three times with sterile PBS and infected with BCG-WT or BCG85B at MOI of 1:5 for 4 h. Thereafter, the monolayer was washed three times with sterile PBS and then overlaid with a 1:10 ratio of BB7-T cell hybridoma (1 × 10^6) specific for Ag85B epitope (aa241–256), which was a kind gift from Dr. Clifford Harding and Dr. Henry Boom, Case Western Reserve University, OH. After 18 h, the cell-free supernatants were collected and tested for IL-2 secretion using an ELISA kit (BD OptEIA, BD Biosciences, San Diego, CA, USA) according to the manufacturer’s protocols.

### *Ex vivo* cytokine analysis

Splenocytes (2.5 × 10^5) from naïve mice and mice immunized with BCG-WT or BCG85B were plated in a 96-well culture plate and stimulated with 20 µg of irradiated whole-cell Mtb lysate for 24 h at 37 °C in 5% CO_2_. The culture supernatants were appropriately diluted and used to measure the concentrations of INF-γ, TNF-α, and IL-2 using an ELISA kit (BD OptEIA, BD Biosciences, San Diego, CA, USA) following the manufacturer’s protocols. Plates were read at 450 nm using a FlexStation 3 plate reader (Molecular Devices).

### Protective efficacy of BCG85B after challenge

Six-week-old C57BL/6 mice (*n* = 5 per group) were immunized subcutaneously with BCG-WT or BCG85B strains as previously described (1 × 10^6 bacilli/100 µl of PBS). Mice in the naïve control group received 100 µL sterile PBS. 30 days post-immunization, mice were challenged with 100 CFUs of Mtb (Erdman) bacteria/mouse using a GlasCol inhalation chamber, as reported previously (32, 102). The mice were humanely sacrificed 30 days post-challenge, and lungs and spleen were extracted, homogenized in sterile PBS containing 0.05% Tween-80, and serially diluted. The dilutions were then plated onto Middlebrook 7H10 agar plates supplemented with OADC and incubated at 37 °C for 3–4 weeks, after which CFUs were counted to evaluate the bacterial burden in each organ. The reduction in CFU counts in vaccinated groups compared to the PBS control group was used as a measure of protective efficacy.

### Statistical analysis

All experiments were performed in triplicate (unless otherwise stated), and data are presented as mean ± SD. Statistical analysis in this study was performed by using Student’s t-test with GraphPad Prism version 10.0 (GraphPad, La Jolla, CA).

## Data Availability

The data presented in this study are deposited in the NCBI Gene Expression Omnibus (GEO) repository under accession number GSE341826.

## Supplementary material

Supplementary Figure 1 Protective effect of BCG85B following immunization and Mtb challenge. **(A)** Schematic representation of immunization and challenge. Mice were immunized on day 0 with BCG or recombinant BCG overexpressing Ag85B (BCG85B). Control animals receive only PBS. On day 30 post-immunization, animals were challenged with Mtb via aerosol infection. On day 30 post-challenge, mice were euthanized, and lungs and spleens were harvested to assess bacterial burden. **(B, C)** Bar graph showing the bacterial load (Colony-forming units (CFU)) in **(B)** lungs and **(C)** spleens of mice from the indicated groups (PBS, BCG, and BCG85B).
